# Effects of Different Aquafeed Sources on Growth Performance, Oxidative Capacity, and Fatty Acid Profile of Three Carps Reared in the Semi-Intensive Composite Culture System

**DOI:** 10.1155/2023/3436607

**Published:** 2023-12-20

**Authors:** Talha Zulfiqar, Muhammad Sajjad Sarwar, Abdul Shakoor Chaudhry, Muhammad Hafeez-ur-Rehman, Mohammed F. El Basuini, Hala Saber Khalil

**Affiliations:** ^1^Department of Zoology, Faculty of Life Sciences, University of Okara, Okara 56300, Pakistan; ^2^Department of Fisheries and Aquaculture, University of Veterinary and Animal Sciences, Lahore, Pakistan; ^3^School of Natural and Environmental Sciences, Agriculture Building, Newcastle University, Newcastle upon Tyne, NE1 7RU, UK; ^4^Animal Production Department, Faculty of Agriculture, Tanta University, 31527, Tanta, Egypt; ^5^Faculty of Desert Agriculture, King Salman International University, South Sinai 46618, Egypt; ^6^Aquaculture Department, Faculty of Fish Resources, Suez University, Suez 43221, Egypt; ^7^College of Fisheries and Aquaculture Technology, Arab Academy for Science, Technology, and Maritime Transport, Alexandria, Egypt

## Abstract

The current experiment is designed to evaluate the effect of different aquafeeds (farm-made versus commercial) on growth, body composition, oxidative capacity, and fatty acid profile in the semi-intensive composite culture system. For this, 1,100 fingerlings/acre having initial body weight and length, *Labeo rohita* (61.34 g, 171 mm), *Catla catla* (71.45 g, 181 mm), and *Cyprinus carpio* (30.80 g, 91 mm) were randomly distributed to 16 ponds and randomly fed on eight different diets (*n* = 2 pond/diet) in a completely randomized research design. Aquafeed were farm-based diets (D1–D2) and commercial aquafeed (D3–D8). The farm-made diets contained various crude protein levels of maize gluten (24.9%) and rice polish (7.3%), whereas commercial diets were procured from commercial feed plants (AMG, Supreme, Aqua, Star Floating, Hi-Pro, and Punjab feed). The growth performance of carps (*L. rohita* and *C. catla*) was significantly improved (*p* < 0.05) by feeding D3 as compared to other diets. Similarly, white blood cell concentration was greater (*p* < 0.05) in all species fed by D3 than in those fed on D7, D8, D5, D6, D1, and D2 fed groups, respectively. Alanine transaminase, aspartate transaminase, and alanine phosphatase activities were significantly lower (*p* < 0.05) in the D3-fed *L. rohita*, *C. catla*, and *C. carpio* compared with those fed on the rest of the treatments. The activities of glutathione peroxidase and superoxide dismutase were also higher (*p* < 0.05) for the D3 fed *L. rohita*, *C. catla*, and *C. carpio* than those fed on the rest diets. The groups fed on D3 and D4 had greater (*p* < 0.05) concentrations of myristic (14), palmitic acid (16), and stearic (18) acids than those fed on the rest of the commercial diets. However, meat chemical composition was similar (*p* > 0.05) across the treatments. These results also prove that the increase in the dietary protein level and lipid content can improve the fish's body's crude protein and fat levels. Feeding D3 improved the production performance, oxidative status, and fatty acid profile in composite major carps culture systems. Thus, based on growth, survival, and body composition, it is concluded that D3 and D4 may be recommended for a commercial culture of major carps. Dietary treatments had no significant impact (*p* > 0.05) on water's physical–chemical properties. Calcium content and alkalinity varied (*p* < 0.05), with D5 showing the lowest calcium and the highest alkalinity.

## 1. Introduction

Aquaculture is known as a stable protein source for human consumption [1, 2]. Major carps are hot climate species that are commonly grown in South Asian countries due to their high-quality meat, lean carcass percentage, and longer shelf life [3, 4]. In modern semi and intensive aquaculture production systems, the major carp are mainly fed on artificial aquafeed, which should be balanced both nutritionally as well as economically to achieve faster growth rates with better production efficiency [5]. In ponds, supplemental feeding provides a quick way to achieve maximum fish output [6]. However, several secondary diet-associated challenges, such as the use of the lower quality ingredients, improper feed formulation, mixing, or pelleting, could result in compromised animal growth, immunity, internal homeostasis disruption, and oxidative stress [7]. It is well established that feeding represents up to 80% of the total cost of production [8]; therefore, feed is known as one of the main important segments in animal production [9]. The production of more than one type of suitable fish at the same time. This is referred to as composite fish culture. In many Asian countries, including Pakistan, this is the most primitive and widely practiced technique [10]. Composite fish culture maximizes fish output from a pond or tank by utilizing all available nutrients in natural niches, supplemented by artificial feeding [11]. The major Indian carps, such as *Labeo rohita*, *Catla catla*, and *Cyprinus carpio*, are the most significant freshwater culturable fishes in Pakistan. Extensive research has been carried out to explore the effects of diverse dietary treatments on the cultivation of major carp species, including *L. rohita*, *C. catla*, and *C. carpio*, within polyculture systems [12–14]. The incorporation of *C. carpio* as a substitute for *C. mrigala* as a bottom feeder has been examined for its impact on the growth performance of major carp species in multiple [15–17]. The goal of semi-intensive polyculture fish culturing is to produce a huge number of fish in a limited time period. Utilization of commercial feed is very important for the development of semi-intensive polyculture of *L. rohita* with other major carps [18].

The artificial fish diet is mostly composed of macronutrients (carbohydrates, protein, and fat) and micronutrients (vitamins and minerals) [19]. Protein is the most significant and expensive ingredient in the fish diet that is obtained primarily from plant or animal sources [20]. It is well accepted that protein directly contributes to the development of living organisms in body structure, tissues, immune system, and metabolism [21, 22]. Several studies were conducted to optimize protein sources and levels in fish diets with varying degrees of success [23–26]. The difference might be due to the new ingredient being studied, which differs from others in terms of dietary content, with an effect on digestibility and feed intake. It is well established that feed processing technologies (grinding and extrusion) result in changes in physical form, nutritional characteristics, and intake of the diet [27]. The quality of the feed varies depending on the ingredients used and how it was processed, which may affect how much of the feed is consumed, how easily the nutrients are absorbed, and how well the cultured organism grows as a result [27]. Further data integrating the information of different diet sources and their influence on the major carp's production is limited. Consequently, the current study aimed to assess the effects of different farm-made and commercial aquafeeds on the growth rate, oxidative capability, and fatty acid profile of three major carps grown in a semi-intensive composite culture system.

## 2. Materials and Methods

### 2.1. Experimental Site and Research Approval

**Figure 1:**
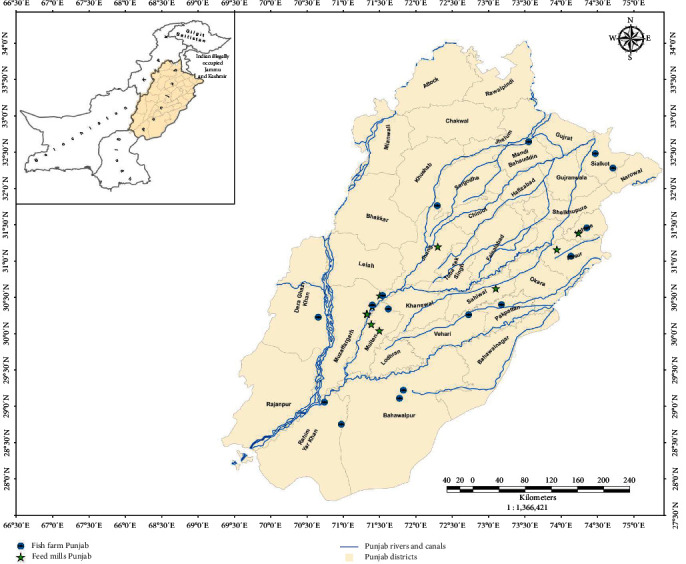
Geographical distribution of fish feed mills and fish farms in Punjab.

All the procedures and protocols of the current research were approved by the Ethical Research Committee of the University of Okara (UO/ERC/2021/15A and 21/01/2021). The current research was conducted in the Department of Fisheries & Aquaculture, University of Veterinary and Animal Sciences, Ravi Campus, C-Block, Pattoki, as well as by collecting feed samples and related information from a number of industrial feed mills, fish seed hatcheries, and several fish ponds in various areas of Punjab, Pakistan, as shown in Figure 1. The proximate analysis was completed at Newcastle University's School of Natural and Environmental Sciences (SNES), situated at the Agriculture Building, Newcastle upon Tyne, United Kingdom.

### 2.2. Research Design and Husbandry Practices

Average 1,100 fingerlings/acre of three carps (800 fingerlings of *L. rohita* and 150 fingerlings of each *C. catla*, *C. carpio* fingerling/specie) were randomized fed one of the eight diets (*n* = 2 pond/diet) then randomly distributed to 16 ponds in a perfectly randomized design. The feeds were farm-based diets (D1–D3) and commercial aqua feeds (D3–D8). The farm-made diets contained various levels of maize gluten (24.9%) and rice polish (7.3%), whereas commercial diets were procured from commercial feed mills (AMG feed, Supreme feed, Aqua feed, Star Floating feed, Hi-Pro feed, and Punjab feed). Dietary ingredients (rice polish and maize gluten) were procured from the local market and analyzed for chemical composition [28], and diets for experimentation have been developed.  Table 1 shows the chemical content of the diets.

Fish were weighed on a monthly basis, and their feed was adjusted up to 2% of the pond's wet mass. To ensure ad libitum consumption, the feed was given two times each day, 08:00 and 16:00 hr. The ponds were filled twice a week and the level of the water was maintained up to five throughout the experiment. Once a week, inorganic and organic manures were applied to the pond to increase its fertility. The 2 weeks' pond acclimatization for fish was followed by a 12-month feeding trial. During the experimental period, aeration was provided consistently to maintain the optimum level of dissolved oxygen and pH, which ranged between 5.7–7.4 and 7.3–8.5 mg/L. The ponds had a wide variety of temperatures between 24.9 and 28.7°C during the feeding trial.

### 2.3. Physiochemical Parameters

The physiochemical parameters were measured on a monthly basis. The temperature and dissolved oxygen of the water were recorded with the help of a dissolved oxygen meter by using (YSI-55/25 FT). pH was measured by a pH meter [29]. Total dissolved solids were measured by a conductivity meter (WTW Cond 330i) and used after setting their range at the “TDS” point [30]. Total alkalinity was measured by the methyl orange indicator method and by using the given formula [31]:

(1) $$\begin{array}{c} \text{Total alkalinity }\left(\text{mg/mL}\right)=\frac{\left(\text{volume of acid used}\right)\left(\text{normality of acid}\right)\left(50,000\right)}{\text{volume of sample }\left(\text{mL}\right)}. \end{array}$$

The carbonates and bicarbonates were estimated by Ellis et al. [32].

(2) $$\begin{array}{c} \text{Carbonates }\left(\text{mg/mL}\right)=\frac{\left(\text{volume of acid used}\right)\left(\text{normality of acid}\right)\left(50,000\right)}{\text{volume of sample }\left(\text{mL}\right)}, \end{array}$$

(3) $$\begin{array}{c} \text{Bicarbonates }\left(\text{mg/mL}\right)=\text{total alkalinity }-\text{carbonates}. \end{array}$$

Total hardness was calculated by Abbas et al. [33].

(4) $$\begin{array}{c} \text{Total hardness }\left(\text{mg/L}\right)=\frac{\left(\text{volume of EDTA used}\right)\times 1,000}{\text{volume of sample }\left(\text{mL}\right)}. \end{array}$$

The amount of Ca^2+^ was calculated by Copaja et al. [34].

(5) $$\begin{array}{c} \text{Calcium }\left(\text{mg/mL}\right)=\frac{\left(\text{volume of EDTA used}\right)\times 1,000}{\text{volume of sample }\left(\text{mL}\right)}. \end{array}$$

The amount of magnesium and total solids were measured after analyzing the total hardness and calcium by Abbas et al. [33] of water, which were estimated by the evaporation method. A 100 mL of water sample was taken in a preweighed beaker and evaporated in an oven at 103°C. After evaporation, beaker was weighed again, and the total solids were calculated by the following:

(6) $$\begin{array}{c} \text{Total solids }\left(\text{mg/mL}\right)=\frac{\text{increase in weight }\times 100,000}{\text{volume of sample }\left(\text{mL}\right)}. \end{array}$$

Total dissolved solids can be measured by a TDS meter (HANNA-HI-98302) and was used after setting its range at the “TDS” point [35].

The salinity of water is measured by a hand refractor meter [36].

### 2.4. Production Performance

Live fish weight and body morphometric measures were taken prior to the feeding trial and again monthly to estimate production performance. The growth performance-associated parameters were calculated by using the following equations:

(7) $$\begin{array}{c} \text{Gain }\left(g\right)=\left(\text{final weight }\left(g\right)-\text{initial weight }\left(g\right)\right), \end{array}$$

(8) $$\begin{array}{c} \text{SGR}=\left(\left(\text{Ln final weight}-\text{Ln initial weight}\right)/\text{days of growth trial}\right)\;\times \;100, \end{array}$$

(9) $$\begin{array}{c} \text{Survival rate }\left(\%\right)=100\times \left(\text{total no. of survived fish/total no. of stocked fish}\right), \end{array}$$

(10) $$\begin{array}{c} \text{Production }\left(\text{kg}/\text{ha}/\text{year}\right)=\left(\left(\text{final biomass }\left(\text{kg}\right)-\text{initial biomass }\left(\text{kg}\right)\right)/\text{water volume }\left(\text{ha}\right)\right). \end{array}$$

### 2.5. Sample Collection

At the feeding trial termination, 20 fishes of each species/pond were randomly selected, weighted, and anesthetized with 150 mg/L tricane methanesulphate (MS-222) according to the protocol of Yildirim-Aksoy et al. [37]. Blood was taken from seven fish from each species using conventional tuberculin needles puncturing the caudal vasculature, and the blood samples were centrifuged at 3,000 × *g* for 15 min to extract the serum. Ethylenediamine tetraacetic acid-coated tuberculin needles were used to puncture the caudal vein, and blood samples were obtained and analyzed for hematological parameters (Automated Hematology Analyzer; MEK6550). Using a commercial kit (21503, Biosystems, Barcelona, Spain), the glucose concentration in the serum samples was measured. The dissection of eight fishes of each species was performed in the sterile laboratory, and organs were collected for biological indices. Five fishes of each species were homogenized (Meat Mincer, ANEX, AG 3060). Blood, meat, and organ samples were obtained and stored at −20°C in labeled plastic zipper bags for further analysis.

### 2.6. Chemical Analysis

The feeds and meat samples were subjected to forced air drying up to 48 hr at 55°C to estimate dry matter contents. These dried samples were crushed and filtered through 1 mm sieve (Foss Grinder, CT 293 Cyclotec, Denmark) and analyzed for crude protein (Method 976.06) and fat contents (Soxhlet procedure, Tecator, Hoganas, Sweden; method 920.29) by following the AOAC (2016) standard procedures. To determine the ash content, samples were burned in a muffle furnace for 3 hr at 620°C.

### 2.7. The Quantities of Thiobarbituric Acid Reactive Substances (TBARS) and Antioxidant Enzymes Essays

TBARS with enzymatic assays were carried out in accordance with the protocol as reported by Mushtaq et al. [4]. To obtain a colorimetric reaction, 1 g sample was homogenized after being added to a 3 mL buffer holding a pH of 7.4 (containing 80 mM tris-maleate and 11.5 g/L KCl). The homogenized sample was then incubated at 37°C for 30 min after being mixed by 1 mL ascorbic acid (2 mM), and finally, 5 mL thiobarbituric acid was boiled. Each sample was then given 5 mL of trichloroacetic acid (200 g/L), centrifuged, and the thiobarbituric acid absorbance measured at 530 nm. The malondialdehyde standard was used to generate a standard curve that was correlated with the sample absorbance readings. An organ sample weighing two grams was homogenized in 6 mL of phosphate buffer (pH 7.4), filtered through Whatman filter paper no. 1, centrifuged at 10,000 × *g* up to 15 min, and enzyme isolation procedures were carried out from the supernatant at 4°C. Superoxide dismutase (SOD), catalase (CAT), as well as glutathione peroxidase (GPx) activity were evaluated in accordance with the prescribed protocol by Mushtaq et al. [4]. Commercial kits were used to determine the aspartate transaminase (AST), alanine transaminase (ALT), and alanine phosphatase (ALP) levels in serum (AL1205, AS3804, AP9764; Randox Laboratories Ltd.).

### 2.8. Fatty Acid Analysis

The extracted fat from liver samples (Soxhlet procedure, Tecator, Hoganas, Sweden; method 920.29) was subjected to gas chromatography (GC) (SHIMADZU, model GC-17A FID) to analyze the fatty acid profile. Fatty acid profiling of the trans-esterified fats was performed through fatty acid methyl esters derivatives (FAME) in accordance with the study by Gecgel et al. [38]. The FAME was analyzed by GC and compared their absolute retention with known standards to identify different groups.

### 2.9. Statistical Analysis

The current research data were analyzed by using SAS's General Linear Model method (Online version) with diets as a fixed factor/independent variable. Means for each fish type were compared by using the Tukey test and declared significant at *p* < 0.05.

## 3. Results

### 3.1. Physiochemical Parameters

The water-associated physiochemical parameters of the ponds enriched with different types of commercial feeds are given in Table 2. There was no influence (*p* > 0.05) of the dietary treatments on the water's physical–chemical properties, including temperature, hardness, and pH. Similarly, magnesium, carbonates bicarbonates, total solids, total dissolved solids, and dissolved oxygen contents did not change (*p* > 0.05) across the groups. However, calcium contents and water alkalinity differed (*p* < 0.05) across the treatments. The D5 group had the lowest calcium contents with the highest alkalinity level than the rest of the treatments.

### 3.2. Growth Performance

During the trial, the survival rate of all groups of *L. rohita*, *C. catla*, and *C. carpio* fed different commercial diets was between 100% and 96%. The initial weight, as well as length of the different fish groups (*L. rohita*, *C. catla*, and *C. carpio*) fed commercial diets were similar (*p* > 0.05) (Table 3). However, different commercial diets significantly influenced (*p* < 0.05) body weight gain, final body weight, specific growth rates (SGR), gain in body length, and final body length of *L. rohita* and *C. catla*. The body weight gain and final body weight were greater in D3 fed carps (*L. rohita* and *C. catla*) in comparison to those carps fed the other aquafeed (*p* < 0.05). Similarly, the final length, gain in body length, and fin lengths were also greater (*p* < 0.05) in D3 fed *L. rohita* and *C. catla* than in the rest of the treatments. Overall, the production performance of *L. rohita* and *C. catla* was improved (*p* < 0.05) by feeding the D3 diet.

### 3.3. Hematological Analysis and Blood Serum Biochemistry

The results for hematology and serum biochemical characteristics of *L. rohita*, *C. catla*, and *C. carpio* fed different commercial diets are given in Table 4. There was a significant change (*p* < 0.05) in means of white blood cells (WBCs), ALT, AST, and ALP among the different dietary treatments. The means for red blood cells, hemoglobin, hematocrit, met hematocrit, and glucose, on the other hand, were similar (*p* > 0.05) between treatments. The WBC concentrations were higher in the D3 fed groups of the carps (*L. rohita*, *C. catla*, and *C. carpio*) than in the D7, D8, D5, D6, D1, and D2 fed groups. The ALT, AST, and ALP activity in the D3-fed *L. rohita*, *C. catla*, and *C. carpio* were substantially lower (*p* < 0.05) than in the other treatments.

### 3.4. Carcass Chemical Composition

The means for carcass chemical analysis of the *L. rohita*, *C. catla*, and *C. carpio* are given in Table 5. The changes in dry matter, ash, protein, and fat content were similar across treatments (*p* > 0.05).

### 3.5. TBARS and Oxidative Capacity Essay

**Figure 2:**
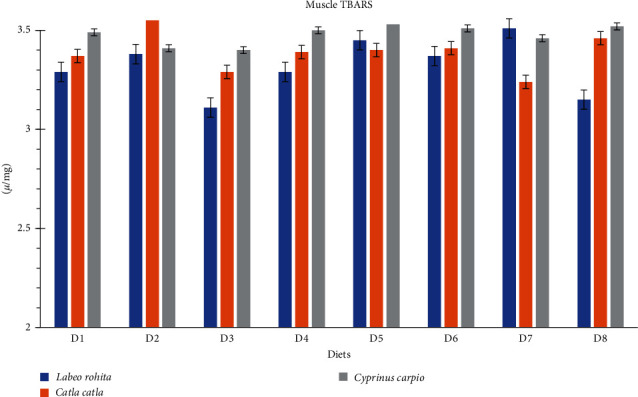
Muscle's TBARS *µ*/mg of different fish species fed on different commercial diets (D1–D8).

**Figure 3:**
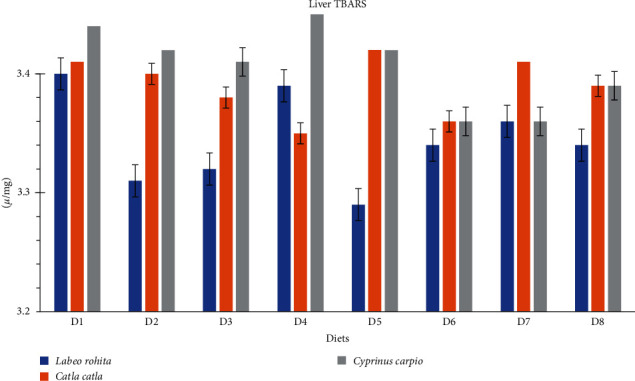
Liver's TBARS *µ*/mg of different fish species fed on different commercial diets (D1–D8).

The results of different antioxidant enzyme activities and TBARS of carps fed on the different commercial feeds are given in Table 6 and Figures 2 and 3. The mean differences of CATs and TBARS in both muscle and liver samples were similar (*p* > 0.05) for *L. rohita*, *C. catla*, and *C. carpio* fed on the different commercial diets. However, dietary treatments affected (*p* < 0.05) the levels of GPx and SOD in both muscle and liver. The activities of SOD and GPx were higher for the D3 fed *L. rohita*, *C. catla*, and *C. carpio* groups than those fed on the rest diets Table 6.

### 3.6. Fatty Acids Analysis

The liver fatty acids profile of *L. rohita*, *C. catla*, and *C. carpio* fed on different commercial diets is given in Table 7. The groups of *L. rohita*, *C. catla*, and *C. carpio* fed on D3 and D4 had greater (*p* < 0.05) concentrations of myristic (14 : 00), palmitic acid (16 : 00), and stearic (18 : 00) acids than those fed on the rest of the commercial diets. However, omega three and six fatty acids such as oleic (18 : 1 *n*−9), linoleic (18 : 2 *n*−6), eicosatetraenoic (20 : 4 *n*−3), and docosahexaenoic (22 : 6 *n*−3) acids amount were similar (*p* < 0.05) across the treatments.

## 4. Discussion

The aquatic environment, diet, and farmed stock are three interlinking factors that alter aquaculture productivity [39, 40]. The cornerstone of sustainable aquaculture is improving these elements [41]. Diet-related parameters such as the source of dietary materials and their inclusion levels can impact the chemical makeup of the diet, influencing feed intake, nutrient utilization, and, ultimately, aquaculture growth performance [27]. It is generally known that an unbalanced diet, particularly in the case of energy and protein content, reduces the growth performance of several animal species [42]. According to the current study's findings, air and water temperatures fluctuated seasonally. The water temperature was somewhat lower than the air temperature throughout the experiment. These findings are in accordance with the result [13, 43], which observed that the water temperature was 2–5°C colder than the air temperature. Additionally, they noted how significantly water temperature affected growth rate, feed consumption, and other metabolic processes. Dissolved oxygen is a crucial element for the development and survival of fish. In all of the treatments, the dissolved oxygen concentration of the pond water stayed within the desirable range of 5.1–8.5 mg/L, which is an ideal range to promote the growth of the fish. As a result of the photosynthetic and respiratory activities, it demonstrated seasonal fluctuation. The hydrogen ion activity (pH) in pond water is an indicator of its environmental status. The pH of the pond water environment changed seasonally throughout the study period due to respiration and photosynthetic activities, with pH values ranging from 7.5 to 8.5 in all treatments. However, statistical analysis revealed a nonsignificant difference between months and treatments. These results are supported by Mahboob and Sheri [44] and Tahir [45]. In all dietary treatments, the pond water stayed alkaline throughout the experiment. The presence of carbonates and bicarbonates causes the pond water to be somewhat alkaline, making it favorable for aquatic organisms [46, 47]. Similar findings were reported by Mahboob and Sheri [44], who observed a positive association between total alkalinity and total hardness as a result of fertilization and additional feed in a carp polyculture system. At the start of the experiment, total solids were at their highest in January and lowest in August. There was a highly significant difference in the months, as well as a substantial difference in the therapies. The presence of total solids and total dissolved solids in pond water promotes the growth of planktonic biomass and contributes to the primary productivity of the pond ecosystem. These findings were consistent with those of [48–50]. The optimum dietary protein level is essential during the developmental stages (larval and fingerlings) of the fish as protein provides biomass for enzymatic synthesis, immune cell formation, muscle elongation, and differentiation. The survival rate of aquatic organisms may be increased by higher protein levels, but often not by the highest ones [51]. In the current experiment, feeding D3 significantly improved the growth performance and the survival rate of carps. These results can be associated with a higher protein content of the D4 diet. Our findings are consistent with Li et al. [52], Yang et al. [26], and Liu et al. [53], as they reported that feeding higher protein dietary levels results in improved production performance of different fish species. Previous researchers [54, 55] and Keshavanath and Gangadhara [56] documented improved performance of polyculture carps fed on a mixed diet of rice bran, cotton seed meal, grinded nut oil cake, and sunflower meal. Azim et al. [57] and Islam et al. [58] similarly reported higher growth performance and survival rates and attained greater biomass of fingerling carp fed on a supplementary diet containing rice bran, soybean meal, and fish meal (40%, 20%, and 10% inclusion on dry basis).

Blood biochemical indices are well-known biomarkers to evaluate the health of the fish [4]. Changes in the concentration of these metabolites identify the metabolic dysfunctionality and injuries of metabolic-associated organs such as the liver [59]. In the current experiment, liver enzymes (ALT, AST, and ALP) were lower for the D3 fed group than the rest of the treatments. These results might be because of the greater availability of amino acid from D3 (due to higher protein contents) for metabolism and the formation of stress-combating proteins. It is well established that increasing protein contents results in greater amino acid availability for enzymatic synthesis and membrane transport activities [60, 61]. The lower enzymatic activities interpreted the positive effects of the D3 diet on major carp metabolism, which resulted in lower stress levels. In our experiment, the WBC counts were higher in the D3 fed group as compared to other treatments. It is shown that WBC produced lysozyme, meaning maximum lysozyme activities [62].

The antioxidant is a primary defense that protects the fish against oxidative stress from different sources, particularly from the environment or endogenous diseases [63]. The current experiment results suggest that the D3 fed group had lower oxidative stress (SOD and GPx activities). These results might be due to the radical scavenging by certain amino acids. The GPx and SOD are present in the cytosol as well as mitochondria of the cell and are mainly involved to protect the cellular organelles against the reactive oxygen species which are produced during cellular respiration (during ATP production) [64]. There were no alterations in CAT activities in the current experiment, which strings above point as it is well established that cytosolic CAT does involve ATP synthesis [64]. The greater GPx activities and SOD show higher aerobic respiration, which results in higher ATP production in D3-fed fish that can justify higher growth performance.

Lipid peroxidation can affect meat quality and shelf life [65]. TBARS test is a well-known diagnostic tool to detect lipid peroxidation [4]. In the current experiment, the values of TBARS were not influenced by different diets. Similarly, the carcass chemical composition was also similar across the treatment groups. The fatty acid profile, on the other hand, was influenced by the different diets. *L. rohita*, *C. catla*, and *C. carpio* fed on D3 and D4 had greater concentrations of myristic (14 : 00), palmitic acid (16 : 00), and stearic (18 : 00) acids than those fed on the rest of the commercial diets. These results can be associated with protein-associated insulin secretion as it is well known that high dietary protein triggers insulin secretion [66]. It has been established that insulin stimulates lipogenesis in trout [67]. However, further research is needed to fully understand these potential causes.

## 5. Conclusion

It was found that the average increase in body weight of (D3–D8) fed with a commercial diet was greater than other farm-made diets (D1–D2). Similarly, the average increase in body length, feed conversion ratio, and SGR of treatments fed with farm-made diets were not recorded good as of commercial diet. Overall, current study results confirmed that optimal dietary protein intake enhanced the production performance, fatty acid profile and antioxidant capacity of major carp. Higher protein diets enhanced GPx and SOD concentrations, which are crucial for activation of the body's antioxidant defense system. The feeding of diets with increased protein contents improved oxidative stress-related hematological parameters, including WBCs and liver health markers (ALT, AST, and ALP).

## Figures and Tables

**Table 1:** Chemical composition of farm-based and commercial diets on a dry basis.

Nutrients	Dietary treatments^1^							
	D1	D2	D3	D4	D5	D6	D7	D8
Dry matter (%)	84.2	84.6	88.7	89.2	87.3	88.2	88.4	88.2
Crude protein (%)	7.3	24.9	26.2	28.3	24.2	22.3	21.9	22.1
Crude fat (%)	3.2	3.2	3.9	3.6	3.4	3.3	3.3	3.3
Crude fiber (%)	7.4	7.3	6.7	6.6	7.3	7.2	7.2	7.2
Ash (%)	9.1	9.1	8.3	8.4	9.1	9.1	9.2	9.1

*Note*. ^1^Dietary treatments = (D1–D8) feeds of different sources.

**Table 2:** Influence of different commercial diets on physiochemical properties of the ponds water.

Parameter	Dietary treatments^1^								SEM^2^	*p*-Value
	D1	D2	D3	D4	D5	D6	D7	D8		
Water temperature (°C)	22.35	22.29	22.25	22.13	22.31	22.14	22.30	22.42	3.502	1.00
Magnesium (mg/L)	40.27	40.02	42.60	41.20	42.25	41.00	41.12	41.87	1.742	0.80
Calcium (mg/L)	21.58^a^	21.46^a^	21.41^a^	21.01^a^	20.63^b^	22.13^a^	23.68^a^	23.58^a^	1.052	0.03
Hardness (mg/L)	218.17	216.08	223.92	217.33	220.58	219.33	219.58	230.08	6.926	0.56
Bicarbonates (mg/L)	377.75	405.88	403.67	402.50	427.46	405.67	399.14	400.70	15.68	0.18
Carbonates (mg/L)	65.83	66.08	64.83	67.08	70.00	71.67	70.00	66.75	4.53	0.76
Alkalinity (mg/L)	443.58^b^	471.96^b^	468.50^b^	469.58^b^	497.46^a^	477.33^b^	467.81^b^	459.33^b^	14.55	0.04
pH	8.29	8.19	8.30	8.22	8.32	8.25	8.17	8.18	0.08	0.46
Dissolved oxygen (mg/L)	6.58	6.96	6.88	6.19	6.92	6.98	6.96	6.86	0.51	0.77
Total solids (mg/L)	1,490.99	1,491.70	1,461.13	1,493.46	1,517.73	1,503.14	1,492.11	1,466.00	25.67	0.41
TDS^3^ (mg/L)	1,383.33	1,376.67	1,352.50	1,371.25	1,393.42	1,389.92	1,400.00	1,385.83	23.20	0.57

*Note*. ^1^Dietary treatments = (D1–D8) feeds of different sources, ^2^SEM = standard error of means, and ^3^TDS = total dissolved solids. ^a−e^Superscripts indicate the significant differences among means within a row.

**Table 3:** Growth performance of major carp species fed different commercial diets.

Parameter		Dietary treatments^1^								SEM^2^	*p*-Value
	Specie	D1	D2	D3	D4	D5	D6	D7	D8		
Body weight (g)											
Initial	*L. rohita*	62.2	61.1	61.3	61.2	60.9	61.3	61.3	61.3	1.17	0.24
	*C. catla*	71.9	71.4	71.3	71.9	71.5	71.4	71.1	71.2	0.96	0.87
	*C. carpio*	30.9	31.1	30.9	31.1	30.4	30.8	30.8	30.4	0.35	0.64
Final	*L. rohita*	1,026.9^d^	1,024.8^d^	1,067.5^a^	1,034.9^c^	1,036.7^c^	1,048.2^b^	1,049.8^b^	1,048.5^b^	2.36	<0.001
	*C. catla*	1,062.1^d^	1,067.8^d^	1,125.3^a^	1,076.8^c^	1,115.5^b^	1,066.1^d^	1,075.6^c^	1,115.3^b^	1.27	<0.001
	*C. carpio*	983.4	985.3	998.7	987.3	983.3	989.4	990.5	991.6	4.85	0.63
Gain	*L. rohita*	964.7^d^	963.8^d^	1006.3^a^	971.8^c^	975.8^c^	989.9^b^	988.5^b^	987.3^b^	1.47	<0.001
	*C. catla*	990.2^d^	996.4^d^	1054.1^a^	1004.9^c^	1044.1^b^	994.8^d^	1004.6^c^	1044.1^b^	5.54	<0.001
	*C. carpio*	952.5	954.3	967.9	956.2	952.9	958.6	959.7	961.2	4.50	0.87
Body length (cm)											
Initial	*L. rohita*	17.1	17.2	17.1	17.1	17.2	17.2	17.1	17.2	0.05	0.11
	*C. catla*	18.1	18.2	18.1	18.1	18.2	18.2	18.2	18.2	0.63	0.29
	*C. carpio*	8.9	9.1	9.2	9.0	9.1	9.1	9.2	9.2	0.15	0.23
Final	*L. rohita*	37.2^c^	37.9^c^	43.3^a^	38.3^c^	40.0^b^	40.0^b^	40.7^b^	41.6^b^	1.26	<0.001
	*C. catla*	31.9^d^	31.3^d^	36.1^a^	32.6^d^	35.1^b^	32.4^c^	31.2^c^	33.1^c^	1.62	0.04
	*C. carpio*	27.7	27.5	28.7	27.6	27.6	27.5	27.7	27.5	0.49	0.09
Gain	*L. rohita*	20.2^c^	20.7^c^	26.2^a^	21.2^c^	22.8^b^	23.9^b^	23.7^b^	23.4^b^	0.79	<0.001
	*C. catla*	23.9^c^	23.1^c^	27.9^a^	24.4^c^	25.1^b^	25.2^b^	25.1^b^	24.9^b^	0.42	<0.001
	*C. carpio*	18.7	18.4	19.4	18.5	18.4	18.4	18.6	18.4	0.47	0.67
Neck fork	*L. rohita*	10.3^c^	10.2^c^	14.5^a^	10.4^c^	11.4^b^	11.4^b^	11.2^b^	11.3^b^	1.03	0.02
	*C. catla*	10.8^c^	10.9^c^	13.6^a^	10.8^c^	11.7^b^	11.5^b^	11.6^b^	11.6^b^	0.99	0.03
	*C. carpio*	9.3	9.2	9.8	9.3	9.2	9.2	9.2	9.2	0.68	0.64
Pectoral fin	*L. rohita*	1.4^c^	1.4^c^	2.5^a^	1.3^c^	1.8^b^	1.9^b^	1.9^b^	1.8^b^	0.022	0.02
	*C. catla*	2.0^c^	2.1^c^	3.1^a^	2.1^c^	2.6^b^	2.7^b^	2.6^b^	2.7^b^	0.02	0.01
	*C. carpio*	0.9	0.9	0.9	0.9	0.9	0.9	0.9	0.9	0.07	0.98
Pelvic fin	*L. rohita*	7.1^c^	7.2^c^	9.1^a^	7.1^c^	7.6^b^	7.6^b^	7.7^b^	7.6^b^	0.02	0.01
	*C. catla*	7.4^c^	7.4^c^	9.36^a^	7.29^c^	7.82^b^	7.85^b^	7.82^b^	7.78^b^	0.052	0.03
	*C. carpio*	6.9	6.8	6.78	6.79	6.89	6.81	6.79	6.82	0.015	0.65
Dorsal fin	*L. rohita*	6.2^c^	6.2^c^	7.01^a^	6.18^c^	6.83^b^	6.81^b^	6.80^b^	6.83^b^	0.021	0.04
	*C. catla*	6.9^c^	6.9^c^	8.25^a^	6.89^c^	7.24^b^	7.27^b^	7.21^b^	7.23^b^	0.034	0.03
	*C. carpio*	5.4	5.4	5.39	5.41	5.46	5.44	5.43	5.40	0.622	0.72
Caudal fin	*L. rohita*	3.1^c^	3.1^c^	4.72^a^	3.03^c^	3.86^b^	3.84^b^	3.83^b^	3.88^b^	0.021	0.03
	*C. catla*	3.3^c^	3.3^c^	4.8^a^	3.39^c^	3.91^b^	3.90^b^	3.91^b^	3.92^b^	0.042	0.01
	*C. carpio*	2.6	2.6	2.6	2.61	2.58	2.66	2.61	2.60	0.720	0.08
Anal fin	*L. rohita*	5.4^c^	5.4^c^	7.2^a^	5.39^c^	5.75^b^	5.76^b^	5.76^b^	5.77^b^	0.051	0.02
	*C. catla*	5.4^c^	5.4^c^	7.3^a^	5.38^c^	5.98^b^	5.89^b^	5.95^b^	5.96^b^	0.022	0.01
	*C. carpio*	4.7	4.8	4.8	4.78	4.81	4.84	4.82	4.79	0.213	0.96
SGR^3^	*L. rohita*	2.9^a^	2.9^a^	3.4^a^	3.02^d^	3.06^d^	3.01^d^	3.21^c^	3.30^b^	0.01	<0.001
	*C. catla*	3.3^e^	3.3^e^	3.9^a^	3.29^e^	3.82^b^	3.28^e^	3.61^d^	3.71^c^	0.01	0.01
	*C. carpio*	0.6	0.6	0.75	0.62	0.59	0.65	0.66	0.68	0.16	0.99
Survival rate (%)	*L. rohita*	98	98	100	97	99	97	97	99	0.34	0.31
	*C. catla*	96	96	100	98	97	100	100	99	0.62	0.43
	*C. carpio*	96	97	99	96	100	96	97	98	0.21	0.63
Production (kg/ha/year)	*L. rohita*	2,074.2	2,087.4	2,297.2	2,076.5	2,176.6	2,175.3	2,167.2	2,189.3	2.012	0.03
	*C. catla*	2,242.8	2,251.2	2,470.6	2,248.1	2,300.2	2,302.4	2,312.2	2,309.4	4.231	0.04
	*C. carpio*	1,976.6	1,963.8	1,967.2	1,951.5	1,964.4	1,962.7	1,949.9	1,976.3	6.934	0.66

*Note*. ^1^Dietary treatments = (D1–D8) feeds of different sources, ^2^SEM = standard error of means, and ^3^SGR = specific growth rates. ^a−e^Means containing different superscripts indicate the significant differences among means within a row.

**Table 4:** Hematology and serum biochemistry of major carp species fed different commercial diets.

Parameter	Specie	Dietary treatments^1^								SEM^2^	*p*-Value
		D1	D2	D3	D4	D5	D6	D7	D8		
Hematology											
WBC (10^6^/*µ*L)^3^	*L. rohita*	476.75^d^	463.59^d^	592.97^a^	496.82^c^	506.10^c^	507.02^c^	534.29^b^	502.07^c^	5.025	0.002
	*C. catla*	465.64^c^	463.42^c^	526.77^a^	475.62^c^	502.97^b^	464.75^c^	463.98^c^	501.40^b^	3.801	0.003
	*C. carpio*	450.66^c^	452.84^c^	556.26^a^	463.20^b^	464.75^b^	463.25^b^	466.52^b^	445.34^b^	2.542	0.003
RBC (10^6^/*µ*L)^4^	*L. rohita*	1.88	1.87	1.91	1.89	2.78	2.68	1.92	1.79	0.952	0.28
	*C. catla*	1.99	1.92	1.98	1.88	1.96	1.78	1.97	1.99	0.920	0.47
	*C. carpio*	1.78	1.76	1.85	1.90	1.79	1.86	1.97	1.91	0.132	0.13
Hb (g/dL)^5^	*L. rohita*	8.94	9.15	8.78	9.02	8.94	8.84	9.05	9.07	0.912	0.23
	*C. catla*	8.83	8.92	9.09	9.01	8.89	8.83	9.00	9.03	0.861	0.12
	*C. carpio*	9.18	8.99	9.08	9.06	9.02	9.09	8.91	8.90	0.752	0.18
HCT (%)^6^	*L. rohita*	36.25	35.11	39.63	38.64	38.36	38.34	39.26	38.99	0.341	0.12
	*C. catla*	32.20	32.27	33.54	34.25	33.97	33.59	32.56	32.43	0.730	0.13
	*C. carpio*	32.36	33.03	33.89	32.98	32.65	32.03	33.12	32.75	0.515	0.26
MCHC (g/dL)^7^	*L. rohita*	26.77	26.26	26.01	26.32	26.72	26.68	26.06	26.40	0.271	0.23
	*C. catla*	26.69	26.64	25.84	26.35	26.48	26.64	26.41	26.48	0.789	0.25
	*C. carpio*	26.92	26.41	26.62	26.44	26.25	26.85	26.28	25.93	0.410	0.40
Serum biochemistry											
ALP (IU/mL)^8^	*L. rohita*	25.40^b^	25.28^b^	19.45^c^	25.35^b^	24.81^b^	26.81^a^	26.56^a^	25.43^b^	0.029	0.01
	*C. catla*	26.24^a^	26.22^a^	23.32^b^	26.43^a^	26.12^a^	26.82^a^	27.03^a^	26.24^a^	0.542	0.03
	*C. carpio*	24.18^a^	23.92^a^	20.73^b^	24.17^a^	23.95^a^	24.12^a^	24.29^a^	24.00^a^	0.431	0.02
AST (IU/mL)^9^	*L. rohita*	74.66^a^	73.13^a^	64.81^b^	73.02^a^	72.24^a^	71.95^a^	73.20^a^	72.79^a^	0.123	0.04
	*C. catla*	73.23^a^	74.21^a^	69.06^b^	73.23^a^	73.61^a^	73.56^a^	72.43^a^	73.03^a^	0.442	0.02
	*C. carpio*	74.16^a^	71.14^a^	65.42^b^	73.55^a^	73.27^a^	72.29^a^	74.18^a^	73.92^a^	0.926	0.03
ALT (IU/mL)^10^	*L. rohita*	47.63^a^	47.38^a^	39.48^b^	48.07^a^	49.61^a^	49.59^a^	47.32^a^	47.03^a^	0.967	0.01
	*C. catla*	51.51^a^	50.23^a^	41.65^b^	49.23^a^	48.16^a^	51.01^a^	48.42^a^	49.56^a^	0.790	0.01
	*C. carpio*	49.79^a^	49.62^a^	40.04^b^	50.28^a^	48.82^a^	49.80^a^	48.26^a^	49.58^a^	0.836	0.03
Glucose (mg/dL)	*L. rohita*	5.84	5.77	5.85	5.71	5.88	5.75	5.63	5.82	0.348	0.32
	*C. catla*	5.72	5.76	5.60	5.85	5.73	5.69	5.61	5.65	0.526	0.09
	*C. carpio*	5.74	5.87	6.68	5.67	5.73	5.71	5.72	5.71	0.451	0.24

*Note*. ^1^Dietary treatments = (D1–D8) feeds of different sources, ^2^SEM = standard error of means, ^3^WBC = white blood cells, ^4^RBC = red blood cells, ^5^Hb = hemoglobin, ^6^HCT = hematocrit, ^7^MCHC = met hematocrit, ^8^ALP = alanine phosphatase, ^9^ALT = alanine transaminase, and ^10^AST = aspartate transaminase. ^a−e^Superscripts indicate the significant differences among means within a row.

**Table 5:** Carcass chemical composition of major carp species fed different commercial diets.

Parameter	Species	Dietary treatments^1^								SEM^2^	*p*-Value
		D1	D2	D3	D4	D5	D6	D7	D8		
Dry matter (%)	*L. rohita*	41.54	41.62	41.23	41.88	40.52	41.41	42.41	40.23	0.162	0.35
	*C. catla*	42.39	41.00	43.53	41.6	43.67	41.89	43.14	43.25	0.354	0.96
	*C. carpio*	42.58	42.39	42.59	42.63	42.5	42.38	42.91	43.56	0.760	0.94
Crude protein (%)	*L. rohita*	16.65	16.62	16.29	16.94	16.88	16.92	16.38	16.89	0.689	0.14
	*C. catla*	16.69	16.33	16.35	16.48	16.32	16.22	16.66	16.87	0.554	0.97
	*C. carpio*	16.52	16.28	16.54	16.57	16.78	16.38	16.78	16.5	0.863	0.60
Fat (%)	*L. rohita*	7.52	7.81	7.61	7.25	7.92	7.88	7.39	7.01	0.907	0.94
	*C. catla*	7.12	7.19	7.22	7.25	7.14	7.34	7.16	7.13	0.968	0.48
	*C. carpio*	7.35	7.62	6.99	7.91	7.67	7.83	7.84	7.83	0.68	0.67
Ash (%)	*L. rohita*	4.79	4.28	4.47	3.79	4.94	4.65	4.70	3.98	0.29	0.65
	*C. catla*	5.55	5.67	5.48	5.23	5.47	5.35	5.40	5.64	0.973	0.64
	*C. carpio*	5.99	6.05	5.68	5.89	6.05	6.10	5.73	5.97	0.935	0.73

*Note*. ^1^Dietary treatments = (D1–D8) feeds of different sources and ^2^SEM = standard error of means. ^a−e^Superscripts indicate the significant differences among means within a row.

**Table 6:** Antioxidant capacity of major carp species fed different commercial diets.

Parameter	Specie	Dietary treatments^1^								SEM^2^	*p*-Value
		D1	D2	D3	D4	D5	D6	D7	D8		
Superoxide dismutase (*µ*/mg)											
Muscle	*L. rohita*	6.71^a^	6.73^a^	6.80^a^	6.74^a^	6.78^a^	6.79^a^	4.32^b^	6.80^a^	0.183	<0.001
	*C. catla*	6.23^a^	6.44^a^	6.23^a^	6.29^a^	6.18^a^	6.29^a^	4.15^b^	6.28^a^	0.123	0.04
	*C. carpio*	6.77^a^	6.76^a^	6.84^a^	6.69^a^	6.76^a^	6.82^a^	5.01^b^	6.79^a^	0.117	0.03
Liver	*L. rohita*	7.05^a^	6.96^a^	7.01^a^	6.97^a^	6.92^a^	6.88^a^	4.19^b^	7.04^a^	0.285	<0.001
	*C. catla*	6.97^a^	6.88^a^	6.91^a^	6.94^a^	6.93^a^	6.88^a^	4.35^b^	6.89^a^	0.102	<0.001
	*C. carpio*	6.98^a^	6.94^a^	6.94^a^	6.90^a^	6.87^a^	6.94^a^	4.17^b^	6.96^a^	0.121	<0.001
Catalases (*µ*/mg)											
Muscle	*L. rohita*	77.28	76.59	75.99	75.99	77.3	77.32	75.26	75.18	1.946	0.12
	*C. catla*	77.47	76.82	76.44	76.73	76.76	78.41	77.14	76.14	1.135	0.40
	*C. carpio*	77.82	75.55	77.98	76.92	75.98	77.33	76.16	75.89	1.323	0.31
Liver	*L. rohita*	78.24	77.57	77.07	76.52	77.81	77.86	76.30	75.42	1.954	0.23
	*C. catla*	77.43	77.56	75.60	77.82	77.8	76.92	77.14	76.71	1.139	0.17
	*C. carpio*	77.9	77.73	77.33	78.7	77.58	78.13	77.46	78.01	1.345	0.32
Glutathione peroxidase (*µ*/mg)											
Muscle	*L. rohita*	256.98^a^	258.60^a^	261.25^a^	267.18^a^	264.39^a^	257.85^a^	221.74^b^	259.35^a^	8.872	0.04
	*C. catla*	250.91^a^	257.64^a^	260.66^a^	258.83^a^	259.44^a^	258.43^a^	220.23^b^	257.26^a^	7.561	<0.001
	*C. carpio*	262.11^a^	269.18^a^	261.62^a^	255.25^a^	261.85^a^	259.46^a^	212.25^b^	256.42^a^	5.251	0.03
Liver	*L. rohita*	259.88^a^	254.12^a^	3.51	251.25^a^	249.94^a^	255.38^a^	227.25^b^	255.74^a^	9.254	0.02
	*C. catla*	234.59^a^	256.93^a^	3.24	258.26^a^	258.10^a^	257.19^a^	215.98^b^	256.64^a^	10.299	0.02
	*C. carpio*	259.93^a^	253.73^a^	3.46	258.73^a^	262.35^a^	251.16^a^	224.35^b^	262.65^a^	7.063	0.04

*Note*. ^1^Dietary treatments = (D1–D8) feeds of different sources and ^2^SEM = standard error of means. ^a−e^Superscripts indicate the significant differences among means within a row.

**Table 7:** Fatty acid analysis of major carp species fed different commercial diets.

Fatty acids codes	Specie	Dietary treatments^1^								SEM^2^	*p*-Value
		D1	D2	D3	D4	D5	D6	D7	D8		
14 : 00	*L. rohita*	3.11^b^	3.10^b^	3.65^a^	3.62^a^	3.12^b^	3.11^b^	3.08^c^	3.10^b^	0.362	0.02
	*C. catla*	3.12^b^	3.13^b^	3.52^a^	3.46^a^	3.14^b^	2.99^c^	2.98^c^	3.19^b^	0.442	0.01
	*C. carpio*	3.23^b^	3.25^b^	3.45^a^	3.38^a^	3.23^b^	3.22^b^	3.14^c^	3.24^b^	0.326	<0.001
16 : 00	*L. rohita*	9.24^b^	9.22^b^	9.37^a^	9.35^a^	9.23^b^	9.22^b^	9.10^c^	9.22^b^	0.434	0.02
	*C. catla*	9.31^b^	9.32^b^	9.49^a^	9.51^a^	9.32^b^	9.29^b^	9.17^c^	9.33^b^	0.524	0.03
	*C. carpio*	9.17^b^	9.15^b^	9.38^a^	9.36^a^	9.15^b^	9.16^b^	9.06^c^	9.15^b^	0.435	0.03
18 : 00	*L. rohita*	5.62	5.61	5.64	5.62	5.62	5.64	5.65	5.64	0.841	0.23
	*C. catla*	5.56	5.55	5.56	5.55	5.54	5.53	5.56	5.55	0.915	0.12
	*C. carpio*	5.92	5.93	5.91	5.93	5.94	5.92	5.90	5.93	0.965	0.11
18 : 1 (*n*−9)	*L. rohita*	11.42	11.42	11.44	11.44	11.45	11.43	11.44	11.45	0.868	0.24
	*C. catla*	11.63	11.64	11.65	11.63	11.59	11.61	11.62	11.63	0.848	0.13
	*C. carpio*	11.62	11.58	11.61	11.59	11.60	11.62	11.59	11.58	0.738	0.31
18 : 2 (*n*−6)	*L. rohita*	14.53	14.56	14.52	14.53	14.56	14.51	14.55	14.52	0.348	0.09
	*C. catla*	13.95	13.96	13.89	13.92	13.88	13.92	13.95	13.96	0.957	0.21
	*C. carpio*	14.56	14.51	14.55	14.53	14.55	14.49	14.52	14.49	0.723	0.16
20 : 4 (*n*−3)	*L. rohita*	4.33	4.35	4.35	4.31	4.33	4.32	4.32	4.33	0.786	0.12
	*C. catla*	4.01	4.01	4.02	4.02	4.05	4.05	4.00	4.06	0.134	0.99
	*C. carpio*	4.17	4.18	4.19	4.16	4.18	4.16	4.17	4.17	0.516	0.62
22 : 6 (*n*−3)	*L. rohita*	15.23	15.26	15.24	15.23	15.23	15.21	15.25	15.28	0.636	0.08
	*C. catla*	14.62	14.65	14.62	14.65	14.62	14.63	14.62	14.59	0.752	0.10
	*C. carpio*	15.69	15.72	15.71	15.69	15.72	15.71	15.72	15.71	0.564	0.21

*Note*. ^1^Dietary treatments = (D1–D8) feeds of different sources and ^2^SEM = standard error of means. ^a−e^Superscripts indicate the significant differences among means within a row.

## Data Availability

The data are available from the first author upon reasonable request.
